# Self assembly of amphiphilic C_60 _fullerene derivatives into nanoscale supramolecular structures

**DOI:** 10.1186/1477-3155-5-6

**Published:** 2007-08-02

**Authors:** Ranga Partha, Melinda Lackey, Andreas Hirsch, S Ward Casscells, Jodie L Conyers

**Affiliations:** 1 Department of Internal Medicine, The University of Texas Health Science Center, Houston, 6431 Fannin St, Houston, TX 77030, USA; 2 Institut für Organische Chemie der Friedrich Alexander Universität Erlangen-Nürnberg, Henkestrasse 42, D – 91054 Erlangen, Germany

## Abstract

**Background:**

The amphiphilic fullerene monomer (AF-1) consists of a "buckyball" cage to which a Newkome-like dendrimer unit and five lipophilic C_12 _chains positioned octahedrally to the dendrimer unit are attached. In this study, we report a novel fullerene-based liposome termed 'buckysome' that is water soluble and forms stable spherical nanometer sized vesicles. Cryogenic electron microscopy (Cryo-EM), transmission electron microscopy (TEM), and dynamic light scattering (DLS) studies were used to characterize the different supra-molecular structures readily formed from the fullerene monomers under varying pH, aqueous solvents, and preparative conditions.

**Results:**

Electron microscopy results indicate the formation of bilayer membranes with a width of ~6.5 nm, consistent with previously reported molecular dynamics simulations. Cryo-EM indicates the formation of large (400 nm diameter) multilamellar, liposome-like vesicles and unilamellar vesicles in the size range of 50–150 nm diameter. In addition, complex networks of cylindrical, tube-like aggregates with varying lengths and packing densities were observed. Under controlled experimental conditions, high concentrations of spherical vesicles could be formed. *In vitro *results suggest that these supra-molecular structures impose little to no toxicity. Cytotoxicity of 10–200 μM buckysomes were assessed in various cell lines. Ongoing studies are aimed at understanding cellular internalization of these nanoparticle aggregates.

**Conclusion:**

In this current study, we have designed a core platform based on a novel amphiphilic fullerene nanostructure, which readily assembles into supra-molecular structures. This delivery vector might provide promising features such as ease of preparation, long-term stability and controlled release.

## Background

Nanotherapeutics has become an increasingly important field of research [1], along with the design and development of novel multifunctional carrier vectors such as nanoparticles [2-4], lipoproteins, micelles, dendrimers [5], nanoshells [6], functionalized nanotubes [7] and polymeric microspheres [8]. Over the past 25 years, conventional phospholipid-based liposomes have been utilized for a variety of biomedical applications ranging from targeted drug delivery [9], diagnostic imaging [10], gene therapy [11] to biosensors [12]. Structural dynamics of the bilayers that constitute liposomal vesicles has been well studied and today, a number of commercially available liposomes are readily used in healthcare applications [13,14]. Liposomes that mimic biological membranes are typically comprised of glycerol-based phospholipids which contain a hydrophilic/polar head-group and one or two hydrophobic/nonpolar hydrocarbon chains of varying length [15]. However in recent years, many other functional artificial nanostructures such as polymeric micelles have been synthesized that offer an alternative choice to phospholipid based liposomes [16]. Carbon-based nanoparticles such as functionalized single-walled carbon nanotubes (SWNTs) and modified C_60 _fullerenes have been the subject of great interest in the last decade because of their potential use in materials, electronics, and, most recently, biological systems [17-19]. Water insoluble fullerene lipid membranes have been designed and well characterized by other groups [20,21].

**Figure 1:**
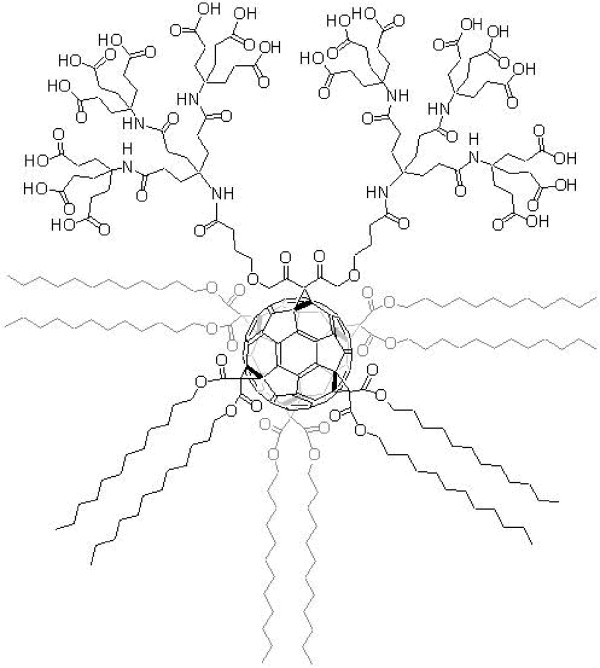
**Chemical structure of the amphiphilic fullerene(AF-1) monomer**. AF-1 readily self assembles into buckysomes. The AF-1 monomer has a molecular weight of 5022 and has six groups attached to the fullerene in an octahedral arrangement with C_2v _symmetry. The functional group at the top of the molecule is a dendritic moiety containing 18 carboxylic acid groups. At the other 5 positions are pairs of C_12 _esters (dodecyl malonates). The pK_a _of the carboxylic acid groups is 7.5 ± 0.127 and thus AF-1 is more soluble at higher pH units [27]. The molecule precipitates out of solution when the pH is less than 3. The average dimension of about 3.5 nm along the main polar axis is similar to that of natural phospho- or glycolipids [24]. In contrast, the typical diameters found in directions perpendicular to this axis are considerably larger that those found for natural double-chain lipids. *(Figure reproduced from reference 24)*.

A novel set of water soluble molecules termed "amphifullerene" compounds have been synthesized by Hirsch and colleagues [22-27]. These amphifullerene nanostructures, based on a C_60 _core, contain both hydrophobic and hydrophilic moieties and self-assemble to form spherical vesicles referred to as "buckysomes" [24]. One such fullerene monomers is AF-1 which consists of a "buckyball" cage to which a Newkome-like dendrimer unit and ten lipophilic C_12 _chains positioned octahedrally to the dendrimer are attached (Figure 1). This globular amphiphile has a low critical micelle concentration and the polar dendrimer head group contains multiple carboxylic acid groups, resulting in pH sensitive assembly and release. The fullerene core in the amphifullerenes acts as an excellent carbon cage to which wide variety of hydrophilic and hydrophobic groups can be attached by well documented methodologies. The fullerene core along with the attached moieties determine the self-assembly process that leads to the formation of different nanostructures [28]. Fullerenes functionalized with different ionic groups have been shown to form aggregates [29], extended nanotubes [30], spheres [28,31,32], and vesicles [33]. Previous models have shown that the molecular volume and length of the chain determines the morphology of the nanostructures that are formed [34]. For example, conical shaped amphiphiles tend to form cylindrical micelles when they have a bulky hydrophilic part and a narrow hydrophobic tail. Stupp and co-workers showed that peptide amphiphiles (PA) of such dimensions have strong electrostatic interactions dominating hydrophobic forces and as a result form long cylindrical micelles termed nanofibers which have potential for manufacturing nanomaterials [35,36]. On the other hand, a variety of amphiphilic dendrimers without fullerene core have been investigated for various biomedical applications [37,38]. Vesicles can carry a higher payload of hydrophilic drugs in their voluminous interiors when compared to most dendrimers. Interestingly, the AF-1 molecule is able to readily self-assembly into both vesicular structures and long cylindrical micelles as shown in this paper. For drug delivery applications, amphiphilic C_60 _fullerenes modified with dendritic moieties and fatty acid side chains are especially attractive due to their potential propensity for vesicle-like self assembly, their ability to encapsulate high payloads of therapeutic molecules, and their tissue specificity when coupled to targeting ligands (i.e., antibodies).

In this current study we have characterized the self assembly of AF-1 using a variety of techniques such as Cryogenic electron microscopy (Cryo-EM), transmission electron microscopy (TEM), and dynamic light scattering (DLS) under varying pH and solvent conditions. The results indicate that AF-1 self assembles readily into both unilamellar and multilamellar vesicles. Cryo-EM results indicate the formation of bilayer membranes with a width of ~6.5 nm, consistent with molecular dynamics simulations [24] for amphifullerenes. We also observe the formation of large (400 nm diameter) multilamellar vesicles and smaller unilamellar vesicles in the size range of 50–150 nm in diameter. In addition, complex networks of cylindrical, rod-like aggregates with varying lengths and packing densities are seen. Other, interesting combined morphologies are also occasionally seen which most likely are transient in nature. The vesicle forming AF-1 (buckysomes) can serve as vehicles for encapsulation of drugs and subsequent drug delivery in a manner similar to liposomes, which have been used for controlled release as well as drug stability, solubility, bioavailability, and reduced toxicity. To utilize the potential application of buckysomes for therapeutic drug delivery we have performed cell viability assays on different human cell lines and have observed no remarkable cytotoxicity. We have also studied the uptake of buckysomes by the cells using fluorescent labelled AF-1 and have imaged the cells using fluorescent microscopy. In summary, this is the first detailed study describing the biophysical characterization, cytotoxicity and bio distribution analysis of the globular amphiphile AF-1.

## Results and Discussion

**Figure 2:**
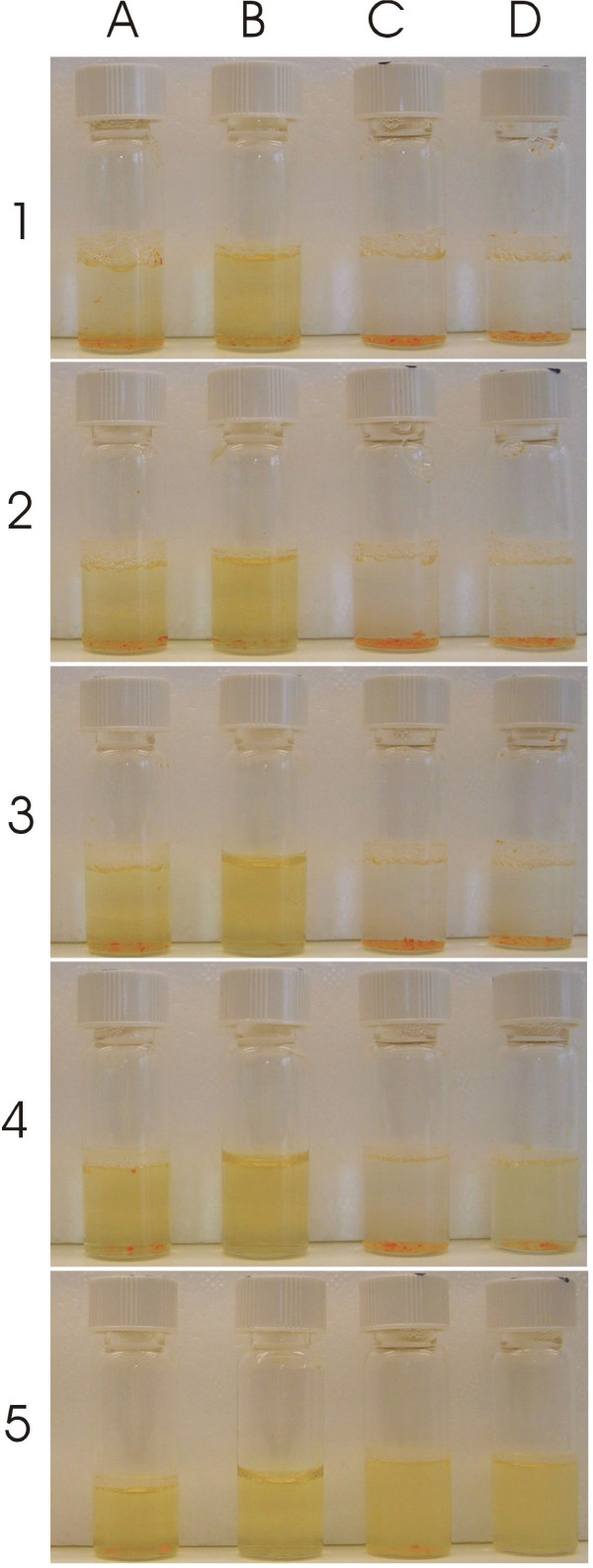
**Solubility profile of AF-1 in varying pH and buffer conditions**. The concentration of AF-1 was kept constant at 2 mg/mL. The buffers were (A) 1 × PBS at pH 7.15, (B) 0.2 M phosphate-citrate at pH 7.0, (C) 10 mM citrate at pH 7.0 and (D) 10 mM citrate at pH 7.4. The time after addition of the buffer was (1) 5 min, (2) 15 min, (3) 30 min, (4, 5) 4 hrs. The vials were gently shaken to disperse AF-1 in solution. However in (5) the sample was vortexed for 5 minutes.

The formation of vesicles by self assembly of AF-1 was reported earlier [23,24]. We investigated this behavior in detail under different aqueous buffers as a function of pH. The polar dendritic group of AF-1 has 18 carboxylic acid groups which provide large number of negative charges per molecule. As a result, variations in pH play a significant role in determining self assembly properties. For biological applications the ideal pH is around 7.0–7.5. At this pH, solubilization of AF-1 can be achieved in PBS (phosphate buffered saline), citrate and phosphate-citrate buffers over a concentration range 0.25 mg/mL to 2.5 mg/mL and using different modes of preparation (simple dispersion, vigorous vortex, extrusion and sonication). However, the extent of solubility varies among the different buffers (Figure 2). AF-1 is readily soluble by dispersion alone in phosphate-citrate buffer at pH 7.0 and fairly soluble in PBS at pH 7.15. In both PBS and phosphate-citrate buffers, a clear yellow solution is obtained that appear stable. In contrast, when AF-1 is hydrated in 10 mM citrate at pH 7.0, it results in producing a turbid solution after vigorous vortexing and standing for 4 hrs. This type of turbidity was not seen in PBS or phosphate-citrate buffer. This turbidity could be an indication of the formation large multilamellar vesicles. We also tested HEPES ((4-(2-hydroxyethyl)-1-piperazineethanesulfonic acid) buffer in the pH range 7.0–8.5. The solubility of AF-1 in HEPES was minimal when compared to the previous three buffers. This was indicated by the presence of insoluble AF-1 powder even after sonication (60–120 mins), heating (up to 95°C) and vigorous vortexing.

### Transmission Electron Microscopy (TEM)

**Figure 3:**
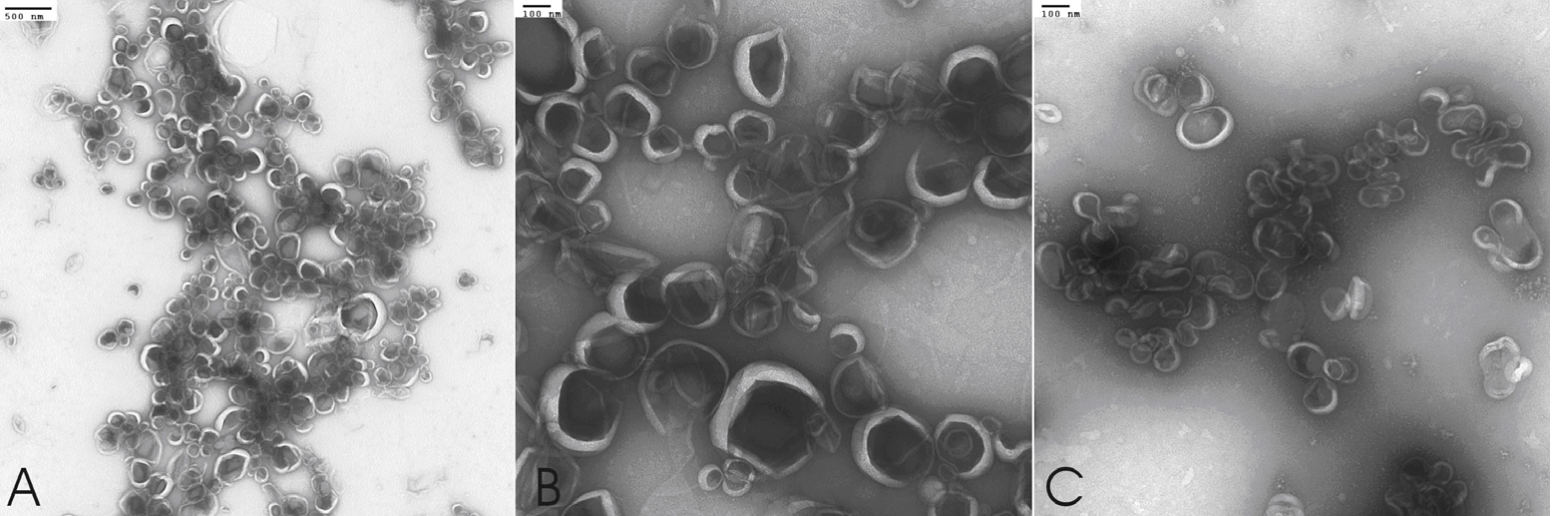
**Uranyl acetate negative stained transmission electron micrographs (TEM) of buckysomes**. The scale bar in (A) is 500 nm and (B, C) is 100 nm. In micrographs (A, B) buckysomes were prepared in 10 mM citrate at pH 7.0 and in (C) Buckysomes were prepared in 1 × PBS buffer at pH 7.15. The concentration of AF-1 was 2 mg/mL and preparations were made at room temperature. Images are representative of 20–30 different areas on the grid.

Negative-stained TEM was performed on AF-1 prepared under various conditions. Figure 3 shows TEM micrographs of AF-1 in citrate and PBS. In the presence of citrate buffer, we observed predominantly vesicles in the size range from 75–100 nm irrespective of the mode of preparation (sonication, vortexing and extrusion), although larger vesicles in the range of 400 nm were occasionally seen as well. Few multilamellar vesicles were clearly seen under these conditions. In the presence of PBS buffer, we also observed 75–100 nm vesicles (Figure 3C), but these were considerably less abundant compared with citrate buffer. Similar results were obtained with other staining agents such as ammonium molybdate and methylamine tungstate, but uranyl acetate provided the best quality stains. We also performed TEM on lyophilized samples, and similar results were seen (data not shown).

### Cryogenic Transmission Electron Microscopy (Cryo-EM)

**Figure 4:**
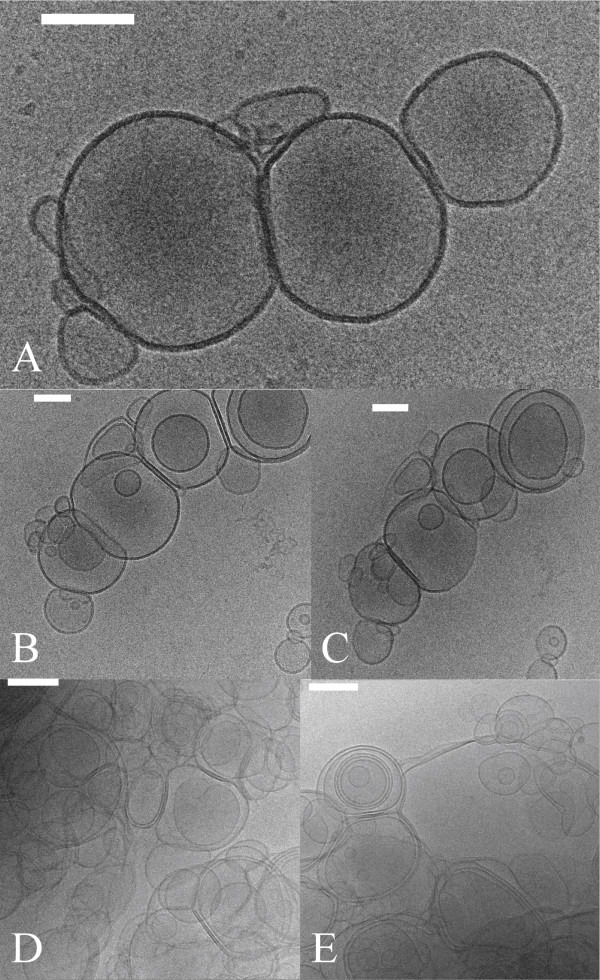
**Representative cryo electron micrographs (Cryo-EM) of buckysomes**. Both unilamellar and multilamellar vesicles are seen. The scale bars in A, B, C, are 100 nm; D, E are 200 nm. Image C is a 45° tilt of B. The bilayer diameter is ~6.5 nm. Buckysomes were prepared in 10 mM citrate at pH 7.0 at a concentration of 2.0 mg/mL. (see Methods for detailed methodology on sample preparation).

Cryo-EM involved freezing the samples in liquid ethane to form vitrified ice. This allows preservation of the vesicles in their native state in contrast with negative-stained preparations. The procedure can be complicated by the fact that some samples produced ice that was too thick for the electron beam to penetrate. The Cryo-EM images in Figure 4 clearly confirm the presence of unilamellar and multilamellar vesicles. The bilayer diameter is ~6.5 nm in agreement with prior results [23,24].

**Figure 5:**
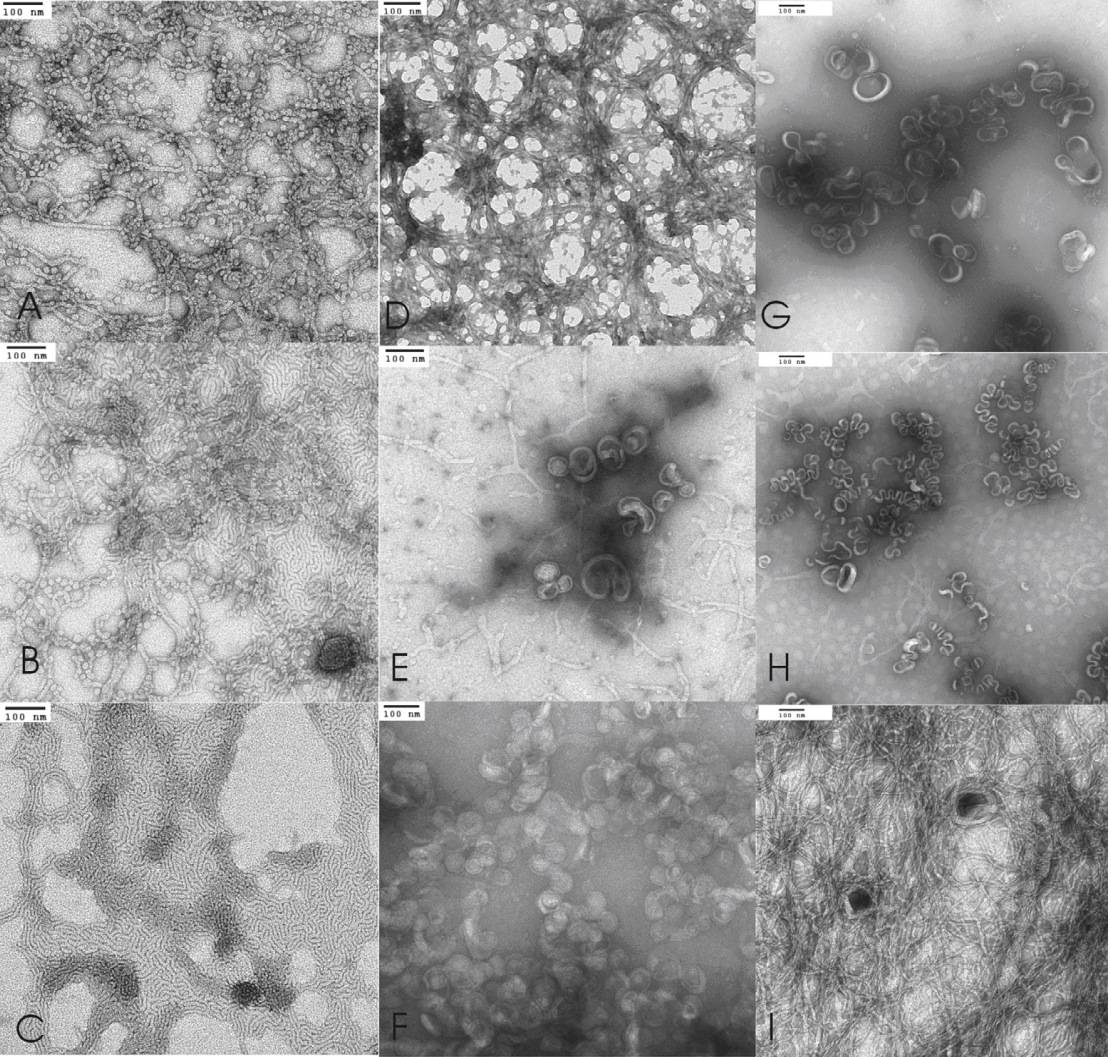
**Uranyl acetate negative-stained transmission electron micrographs (TEM) of various supramolecular structures of AF-1**. Combined morphologies of rod-like, branched and elongated micelles are seen in addition to buckysomes. The scale bar is 100 nm in all the images. In micrographs (A, B, C) AF-1 was prepared in 10 mM HEPES at pH 8.0; in (D, E, F) AF-1 was prepared in 0.2 M phosphate-citrate and in (G, H, I) in 1 × PBS buffer at pH 7.15. The concentration of AF-1 was 2 mg/mL and preparations were made at room temperature. Images are representative of 20–30 different areas on the grid.

Using both negative-stained TEM and Cryo-EM we observed, in addition to vesicles, other interesting supramolecular structures as well that vary with pH. TEM of structures formed in HEPES buffer demonstrate predominantly rod-like structures (Figure 5A, 5B) at pH 8 or higher, whereas at pH 7.5 and below spherical vesicles are seen as well. The rod-like elongated micelles have a diameter of ~6.5 nm which is consistent with the bilayer arrangement seen in vesicles. Other self-assembled structures formed under these conditions resemble worm-like micelles (Figure 5C). These structures are very similar to asymmetric amphiphilic diblock copolymers that self assemble in selective solvents [39]. In both phosphate citrate (Figure 5D, E, F) and PBS (Figure 5G, H, I) buffers, we observe a mixture of vesicles and elongated micelles. Comparable results demonstrating the presence of rod-like and worm-like structures were seen in cryo-TEM micrographs (data not shown). Studies on the self assembly of certain surfactants have described the interplay of theoretical and physical parameters that control the formation of vesicles and micelles [40].

In this case, there is a complex interplay between three major factors namely the (a) charges on the carboxylic acid groups present in the dendrimer which is controlled by the pH, (b) the solvation process (affected by the solvent) and (c)the mode of preparation (sonication or vortexing). These three critical parameters determine whether the end self-assembly structure is a vesicles or a long cylindrical micelle. At pH higher than 7.5 and the presence of HEPES buffer, the cylindrical micelles seemed to be the favoured structure irrespective of the mode of preparation. At pH 7.0 with citrate buffer as the solvent, vesicles are present. Since both the structures are formed from the same AF-1 molecule, the effect of chain length affecting the morphology as described in several papers does not come into play [28]. However, it is well evident that 10 mM citrate in the pH range 7.0–7.4 is necessary for forming the vesicles (Figure 3 &4). When phosphate was added to citrate at the same pH range, mixed morphologies are seen (Figure 5D). In an earlier study, Tour and co-workers reported the effect of solvent polarity as a factor affecting the folding of side-chains resulting in both nanorods and vesicles from the same C_60 _derivative [41]. The effect of the solvent on the environment around the AF-1 molecule seems to be the key factor governing the formation of different nanostructures at a given pH and preparation methodology. This present study focuses on describing the novel structures observed upon self-assembly of amphifullerenes as well as their biological behaviour. Future studies will be aimed at understanding the driving forces that determine the formation of a specific self assembled structure.

### Dynamic Light Scattering (DLS)

**Figure 6:**
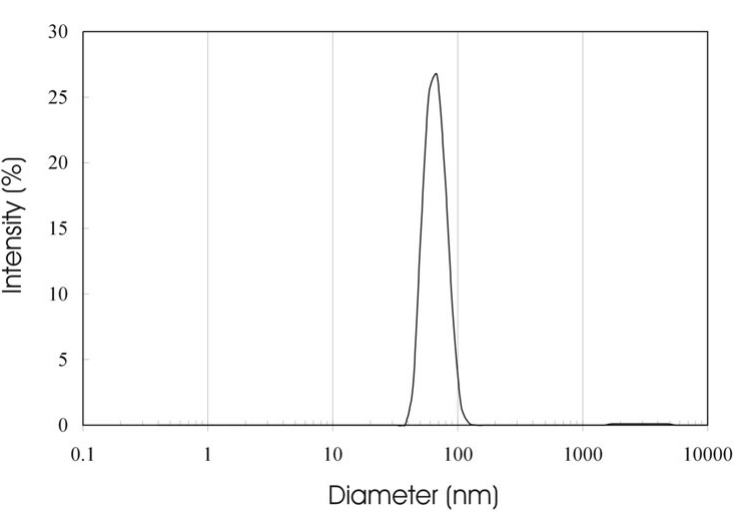
**Size characterization of buckysomes using Dynamic light scattering (DLS)**. Size distribution by DLS of buckysomes (2.0 mg/mL) prepared at pH 7.0 in 10 mM citrate buffer. The AF-1 dry powder was hydrated for 30 minutes at room temperature in the buffer and then extruded at 100°C using a 100 nm polycarbonate membrane. The average hydrodynamic diameter of the vesicles is 68 nm after 5 measurements. The correlation coefficient against time (μs) was fitted by a CONTIN algorithm in a multimodal fit. The size distribution ranges from 50 nm to 80 nm for the vesicles. The zeta potential in 10 mM citrate at pH 7.0 was -48 mV.

DLS results are based on the assumption that particles are spherical in nature. However, since we see a mixture of both spherical vesicles and rod-like elongated micelles in certain cases, the interpretation of the DLS results is difficult at best. In most cases, the polydispersity index (PdI) is higher than normal values, making it difficult to analyze the data. However, in certain instances (Figure 6), a sharp peak with a 68 nm average diameter value is observed with a PdI of 0.08. In this particular case, AF-1 (2 mg/mL) was prepared by extrusion at high temperatures (100°C) using a 100 nm polycarbonate membrane in 10 mM citrate at pH 7.0. However, a similar size-distribution profile has been observed using citrate buffer under other preparation conditions as well. We also compared DLS measurements of AF-1 prepared in HEPES, PBS, citrate and phosphate-citrate buffers. Concentrations of AF-1 for these experiments ranged from 0.25 mg/mL to 3.0 mg/mL and the pH was varied from 6.5 to 9.0. Different modes of preparation were used to solubilize AF-1 in cases where solubility was limited. The results of DLS were inconclusive in all these cases due to high PdI and a wide size peak (data not shown). One possible explanation could be the presence of a mixture of spherical vesicles with different sizes.

### Cytotoxicity and cellular localization

**Figure 7:**
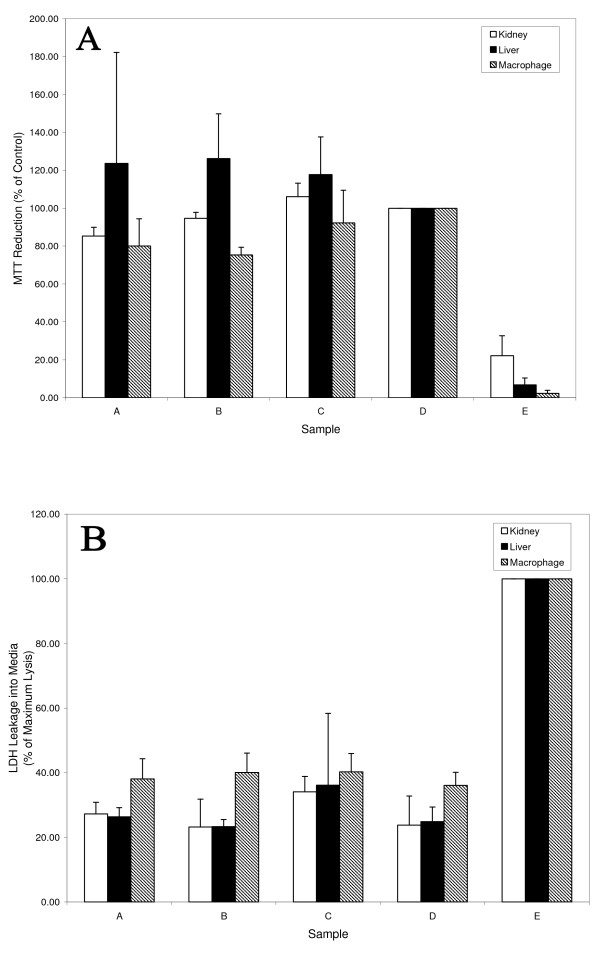
**MTT and LDH assays**. (A) MTT and (B) LDH assay showing the effects of buckysomes on cell viability and proliferation. Kidney, Liver, and Macrophage cells exhibited little differences when compared to PBS controls after exposure to AF-1 at different concentrations and analyzed for membrane integrity (LDH) as well as cellular proliferation (MTT). Samples A, B, C, D and E are 2 mg/mL AF-1, 0.2 mg/mL AF-1, 0.02 mg/mL AF-1, cells only, and control respectively. Cells were treated with 0.1% H_2_O_2 _for negative control of MTT and 0.9% Triton X-100 for positive control of LDH.

**Figure 8:**
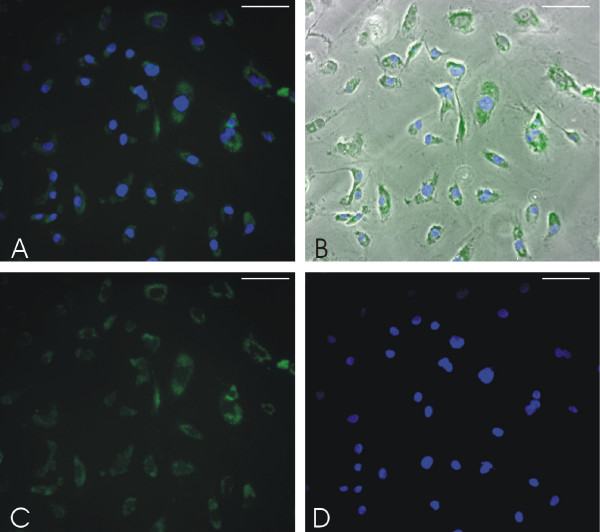
**Fluorescent microscopy of human coronary artery endothelial cells incubated with 6-aminofluorescein-buckysomes for 18 hrs**. The fluorescein coupled buckysomes were clearly cell associated, with no change in localization following several washes with PBS. Cells were fixed and counterstained with DAPI. (A) Superimposed image of fluorescein and DAPI emission. (B) Panel A superimposed with bright field image of cells. (C) Fluorescein emission at 520 nm. (D) DAPI emission at 461 nm. The scale bar for all panels is 50 μm.

The formation of vesicles by AF-1 under specific conditions opens up possibilities for applications in drug delivery. In order to determine the effects of AF-1 on cell proliferation and cytotoxicity, we conducted in vitro MTT dye reduction assays and LDH release assays on several human cell lines (Figure 7). Fluorescein-labelled AF-1 was used to observe the cellular association of AF-1 vesicles in human coronary artery endothelial cells (Figure 8). For cellular toxicity studies, AF-1 vesicles were prepared by vortexing in 10 mM citrate at pH 7.0 followed by conjugation with Fluorescein (see Methods). The presence of the structures was confirmed with Transmission Electron microscopy. The fluorescent micrographs clearly show that the AF-1 vesicles are cell associated. Most of the cells showed strong fluorescence intensity in all areas except the nucleus. The cells did not show any morphological changes when compared to control cells incubated with PBS. Future experiments using confocal microscopy can confirm the intracellular localization of these AF-1 vesicles.

## Conclusion

Self assembly of molecules in the nano-scale is of great interest due to their potential in biomedical applications. In this present study we have investigated the biological role of a novel globular amphiphile (AF-1) with a fullerene core, a dendrimeric polar head-group and hydrophobic tails mimicking conventional phospholipids. The modified water soluble fullerene core could serve as a template for easy linking of different drug molecules. Currently we are analyzing the conditions needed for the critical tuning of several variables that determine homogenous distribution of selective morphologies. The different factors are pH, sample concentration, temperature, type of dispersant and method of preparation. The results could provide clues for synthetic modifications on the monomer structure to tailor specific nanostructures. In the future, we are planning to perform *in vivo *experiments of antibody linked buckysomes loaded with contrast agents for targeted diagnostic imaging of vulnerable plaque.

## Methods

### (a) Buckysome Preparation

The globular amphiphile AF-1 was synthesized as previously described [24]. The buckysome preparation was carried out by either one of the four different approaches namely: (a) simple hydration in buffer with occasional shaking to remove clumps, (b) vigorous vortex, (c) sonication for 15 min using a Branson 3510 sonicator and (d) heating followed by extrusion through a mini-extruder (Avanti Polar Lipids, Alabaster, AL) using a 100 nm polycarbonate filter. Extrusion was performed for a total of 21 passes (back and forth). The resulting suspension was analyzed by Cryo-EM, negative stained TEM and DLS.

Buckysomes were coupled to 6-aminofluorescein (Fluka-Sigma-Aldrich, St. Louis, MO) using the following procedure. 400 μL of buckysomes (2 mg/mL) was incubated with 100 μL each of 0.25 M EDC (*N*-Ethyl-*N'*- [3- dimethylaminopropyl]carbodiimide) (Fluka) and 0.25 M sulfo-NHS (N-hydroxysulfosuccimide) (Pierce, Rockford, IL) for 2 hrs at room temperature. The pH was adjusted to 7.0 using NaOH. To this solution, 300 μL of 6-aminofluorescein (1 mg/mL prepared in DMSO) was added and incubated overnight at room temperature. The free 6-aminofluorescein was separated from 6-aminofluorescein coupled AF-1 by size exclusion chromatography on Sephadex^® ^G-75 (Sigma-Aldrich, St. Louis, MO) column. The fractions were analyzed by fluorometry (Tecan Systems Inc, San Jose, CA) for 6-aminofluorescein emission at 520 nm.

### (b) Transmission Electron Microscopy

The buckysomes were visualized using uranyl acetate negative staining. A 400 mesh Copper grid coated with Carbon film and stabilized with Formvar (Ted Pella Inc, Redding, CA) was coated with poly-L-Lysine prior to the sample staining. The sample was placed on the grid for 5 minutes and excess of sample was blotted with filter paper. The samples were stained with 1% solution of uranyl acetate for 1 minute and allowed to dry. Analysis of the stained grids was performed with a JEOL JEM-1010 Transmission Electron Microscope (Tokyo, Japan) at an accelerating voltage of 80 kV. The images were captured with the AMT Advantage digital CCD Camera system.

### (c) Cryo-Electron Microscopy

A 5 μL drop of the buckysome was frozen in liquid ethane on a holey carbon copper grid coated with ultrathin 3 nm carbon (Ted Pella Inc, Redding, CA). Vitrobot™ (FEI, Holland) was used for automated cryo freezing of the grids (1 sec hang time, 1 blot, room temperature). The data were collected with a TVIPS (Gauting, Germany) F415 4 K × 4 K slow-scan CCD camera on a FEI (Eindhoven, Holland) Tecnai G^2 ^TF30 Polara electron microscope operating at 300 kV and at liquid nitrogen temperature by using low-dose protocol. The post magnification value was 1.615 and the CCD pixel size was 15 microns. The micrographs were processed with EMAN v1.7 software (Baylor College of Medicine, Houston, TX).

### (d) Dynamic light scattering

Dynamic light scattering (DLS) measurements were performed using a Malvern Nano-ZS zetasizer (Malvern Instruments Ltd, Worcestershire, United Kingdom). The Nano-ZS employs non-invasive back scatter (NIBS™) optical technology and measures real time changes in intensity of scattered light as a result of particles undergoing Brownian motion. The sample is illuminated by a 633 nm Helium-Neon laser and the scattered light is measured at an angle of 173° using an avalanche photodiode. The size distribution of the vesicles is calculated from the diffusion coefficient of the particles according to Stokes-Einstein equation. The average diameter and the polydispersity index of the samples are calculated by the software using CONTIN analysis.

### (e) Zeta potential measurements

The zeta potential of liposomes was measured with the Malvern Nano ZS using the technique of Laser Doppler Velocimetry (LDV). In this technique, a voltage is applied across a pair of electrodes at either end of the cell containing the particle dispersion. Charged particles are attracted to the oppositely charged electrode and their velocity was measured and expressed in unit field strength as an electrophoretic mobility. The zeta potential was calculated from the electrophoretic mobility using Henry's equation (Hunter, R. J.*Zeta Potential in Colloid Science, Principles and Applications, Academic Press, London*, 1981).

### (f) Cell Culture

Human Kidney Epithelial cells (CC-2556) and Human Coronary Artery Endothelial cells (CC-2585 were obtained from Cambrex Corp. (Baltimore, MD). Kidney cells were grown in REGM media supplemented with REGM BulletKit^® ^(Cambrex). Endothelial cells were grown in EBM media supplemented with EGM-2 BulletKit^® ^(Cambrex). HepG2 Liver Hepatocellular Carcinoma cells (HB-8065) and Murine Macrophage-like Cells (TIB-67) were obtained from American Type Culture Collection (Manassas, VA). HepG2 cells were grown in Earle's Minimal Essential Media (ATCC) supplemented with 10% fetal bovine serum (Gibco^®^, Invitrogen, Carlsbad, CA), 2 mM L-glutamine, 100 μg/mL penicillin and 100 U/mL streptomycin (Sigma-Aldrich, St. Louis, MO). Macrophages were grown in Dulbecco's Modified Eagle's Medium (ATCC) supplemented with 10% fetal bovine serum (Gibco^®^), 2 mM L-glutamine, 100 μg/mL penicillin and 100 U/mL streptomycin (Sigma-Aldrich). All cells were grown at 37°C in 5% CO_2_.

### (g) Cytotoxicity

Murine Macrophage-like cells (MAC, ATCC); HepG2 Liver cells (LIV, ATCC); and Human Kidney Epithelial Cells (HKEC, Cambrex) were exposed to varying concentrations of buckysomes for 18 hrs at 37°C, 5%CO_2_. Cells were then analyzed for general cytotoxicity using 3-(4,5-Dimethylthiazol-2-yl)-2,5-diphenyltetrazolium bromide (MTT) and Lactate Dehydrogenase (LDH) assays from Roche Applied Sciences (Indianapolis, IN) and Promega (Madison, WI) respectively.

#### LDH Assay

Leaking membranes of damaged or dead cells release the cytoplasmic enzyme lactate dehydrogenase (LDH) into the surrounding media. This enzyme can be detected by measuring its catalytic activity and indirectly the conversion of 2-(4-Iodophenyl)-3-(4-nitrophenyl)-5-phenyl-2H-tetrazolium chloride (INT) to another water-soluble formazan dye. Briefly, 2.5 × 10^4 ^viable cells were seeded in black-walled Falcon 96 well tissue culture-treated microtiter plates and allowed to attach overnight at 37°C/5%CO_2_. Cells were then inoculated with appropriate concentrations of AF-1 or control materials and incubated for 18 hrs at 37°C/5% CO_2_. The LDH assay was performed using the Cyto-Tox ONE™ Membrane Integrity Assay (Promega, Madison, WI) according to the manufacturer's instructions. Results were given as relative values to cells treated with 0.9% Triton-X (vol:vol). Cells only control was treated with equal volumes of Dulbecco's phosphate buffered saline.

#### MTT Assay

For each set, 2.5 × 10^4 ^viable cells were seeded into wells of a Falcon 96-well tissue culture-treated microtiter plate (Becton Dickenson, Franklin Lakes, NJ) in triplicate. Cells were treated with the described particle suspensions in a concentration of 50 μg/mL in complete culture medium for 24 hr. Cytotoxicity was determined by measuring the reduction of the water-soluble MTT (3-(4,5-Dimethyl-2-thiazolyl)-2,5-diphenyl-2H-tetrazolium bromide, SIGMA) molecule to water-insoluble MTT-formazan, after incubating in 100 μL solubilization buffer for 24 hr at 37°C/5% CO_2_. The wells are then measured for absorbance at 550 nm using a Safire^2^™ plate reader (Tecan Systems Inc, San Jose, CA). The results are given as relative values to cells treated only with equal volumes of Dulbecco's phosphate buffered saline.

### (h) Localization of 6-aminofluorescein conjugated AF-1 using Fluorescence Microscopy

Human Coronary Artery Endothelial Cells (Cambrex) were grown in 8-chamber tissue culture slides and exposed to 6-aminofluorescein-buckysomes for 18 hrs at 37°C, 5%CO_2_. After two washes with Dulbecco's phosphate buffered saline (Gibco^®^), cells were fixed in 4% paraformaldehyde (Sigma-Aldrich) for 20 min, and washed twice with Dulbecco's phosphate buffered saline. Chambers were removed and slides were dried. Fixed cells were mounted in ProLong^® ^Gold antifade reagent with DAPI (4',6-diamidino-2-phenylindole) (Invitrogen, Carlsbad, CA). Images of fixed cells were taken with an Olympus IX71 inverted microscope (Olympus America Inc, Center Valley, PA) and Retiga 2000R Camera (Q Imaging, Burnaby, BC, Canada). Images were processed using Compix SimplePCI software (Compix Inc, Sewickley, PA).

## Competing interests

The author(s) declare that they have no competing interests.

## Authors' contributions

Please see sample text in the instructions for authors. RP and ML performed the experiments. RP and JLC designed the overall project and wrote the manuscript, with inputs from AH and SWC towards the final draft. All authors read and approved the final manuscript.

## References

[B1] Ferrari M (2005). Cancer nanotechnology: opportunities and challenges. Nat Rev Cancer.

[B2] Torchilin VP (2006). Multifunctional nanocarriers. Adv Drug Deliv Rev.

[B3] Gregoriadis G, (Editor) (1988). Liposomes as Drug Carriers.

[B4] Rolland A, (Editor) (1993). Pharmaceutical Particulate Carriers.

[B5] Joester D, Losson M, Pugin R, Heinzelmann H, Walter E, Merkle HP, Diederich F (2003). Amphiphilic dendrimers: novel self-assembling vectors for efficient gene delivery. Angew Chem Int Ed Engl.

[B6] Portney NG, Ozkan M (2006). Nano-oncology: drug delivery, imaging, and sensing. Anal Bioanal Chem.

[B7] Klumpp C, Kostarelos K, Prato M, Bianco A (2006). Functionalized carbon nanotubes as emerging nanovectors for the delivery of therapeutics. Biochim Biophys Acta.

[B8] Barratt G (2003). Colloidal drug carriers: achievements and perspectives. Cell Mol Life Sci.

[B9] Freeman AI, Mayhew E (1986). Targeted drug delivery. Cancer.

[B10] Seltzer SE (1989). The role of liposomes in diagnostic imaging. Radiology.

[B11] Lasic DD, Papahadjopoulos D (1995). Liposomes revisited. Science.

[B12] Tien HT, Salamon Z, Ottova A (1991). Lipid bilayer-based sensors and biomolecular electronics. Crit Rev Biomed Eng.

[B13] Porche DJ (1996). Liposomal doxorubicin (Doxil). J Assoc Nurses AIDS Care.

[B14] Boswell GW, Buell D, Bekersky I (1998). AmBisome (liposomal amphotericin B): a comparative review. J Clin Pharmacol.

[B15] Litman BJ (1973). Lipid model membranes. Characterization of mixed phospholipid vesicles. Biochemistry.

[B16] Nishiyama N, Kataoka K (2006). Current state, achievements, and future prospects of polymeric micelles as nanocarriers for drug and gene delivery. Pharmacol Ther.

[B17] Lacerda L, Bianco A, Prato M, Kostarelos K (2006). Carbon nanotubes as nanomedicines: from toxicology to pharmacology. Adv Drug Deliv Rev.

[B18] Bianco A, Kostarelos K, Partidos CD, Prato M (2005). Biomedical applications of functionalised carbon nanotubes. Chem Commun (Camb).

[B19] Bianco A, Kostarelos K, Prato M (2005). Applications of carbon nanotubes in drug delivery. Curr Opin Chem Biol.

[B20] Murakami H, Watanabe Y, Nakashima N (1996). Fullerene lipid chemistry: Self-organized multibilayer films of a C_60_-bearing lipid with main and subphase transition. J Am Chem Soc.

[B21] Nakanishi T, Morita M, Murakami H, Sagara T, Nakashima N (2002). Structure and electrochemistry of self-organized fullerene-lipid bilayer films. Chemistry.

[B22] Brettreich M, Hirsch A (1998). A highly water-soluble dendro[60]fullerene. Tetrahedron Lett.

[B23] Brettreich M, Burghardt S, Bottcher C, Bayerl T, Bayerl S, Hirsch A (2000). Globuläre amphiphile: membranbildende Hexaaddukte von C_60_. Angew Chem.

[B24] Brettreich M, Burghardt S, Bottcher C, Bayerl T, Bayerl S, Hirsch A (2000). Globular amphiphiles: membrane-forming hexaadducts of C(60). Angew Chem Int Ed.

[B25] Burghardt S, Hirsch A, Schade B, Ludwig K, Bottcher C (2005). Switchable supramolecular organisation of tructurally defined micelles based on an amphiphilic fullerene. Angew Chem Int Ed.

[B26] Braun M, Atalick S, Guldi DM, Lanig H, Brettreich M, Burghardt S, Hatzimarinaki M, Ravanelli E, Prato M, Van Eldik R, Hirsch A (2003). Electrostatic complexation and photoinduced electron transfer between Zn-cytochrome c and polyanionic fullerene dendrimers. Chem Eur J.

[B27] Maierhofer AP, Brettreich M, Burghardt S, Vostrowsky O, Hirsch A, Langridge S, Bayerl TM (2000). Structure and electrostatic interaction properties of monolayers of amphiphilic molceules derived from C_60 _fullerenes: A film balance, neutron- and infrared reflection study. Langmuir.

[B28] Guldi DM, Zerbetto F, Georgakilas V, Prato M (2005). Ordering Fullerene Materials at Nanometer Dimensions. Acc Chem Res.

[B29] Angelini G, De Maria P, Fontana A, Pierini M, Maggini M, Gasparrini F, Zappia G (2001). Study of the Aggregation Properties of a Novel Amphiphilic C_60 _Fullerene Derivative. Langmuir.

[B30] Gan HY, Liu HB, Li YL, Gan LB, Jiang L, Jiu TG, Wang N, He XR, Zhu DB (2005). Fabrication of fullerene nanotube arrays using a template technique. Carbon.

[B31] Liu Y, Xiao SQ, Li HM, Li YL, Liu HB, Lu FS, Zhuang JP, Zhu DB (2004). Self-assembly and characterization of a novel hydrogen-bonded nanostructure. J Phys Chem B.

[B32] Georgakilas V, Pellarini VF, Prato M, Guldi DM, Melle-Franco M, Zerbetto F (2002). Supramolecular self-assembled fullerene nanostructures. Proc Natl Acad Sci.

[B33] Zhou S, Burger C, Chu B, Sawamura M, Nagahama N, Toganoh M, Hackler UE, Isobe H, Nakamura E (2001). Spherical bilayer vesicles of fullerene-based surfactants in water: a laser light scattering study. Science.

[B34] Israelachvili JN, Mitchell DJ, Ninham BW (1977). Theory of self-assembly of lipid bilayers and vesicles. Biochim Biophys Acta.

[B35] Hartgerink JD, Beniash E, Stupp SI (2002). Peptide-amphiphile nanofibers: a versatile scaffold for the preparation of self-assembling materials. Proc Natl Acad Sci.

[B36] Hartgerink JD, Beniash E, Stupp SI (2001). Self-assembly and mineralization of peptide-amphiphile nanofibers. Science.

[B37] Lee CC, MacKay JA, Frechet JMJ, Szoka FC (2005). Designing dendrimers for biological applications. Nat Biotech.

[B38] Stiriba SE, Frey H, Haag R (2002). Dendritic Polymers in Biomedical Applications: From Potential to Clinical Use in Diagnostics and Therapy. Angew Chem Int Ed.

[B39] Cameron NS, Corbierre MK, Eisenberg A (1999). E. W. R. Steacie award lecture, Asymmetric amphiphilic block copolymers in solution: a morphological wonderland. Can J Chem.

[B40] Jan Engberts BFN, Kevelam J (1996). Formation and stability of micelles and vesicles. Current opinions in colloid and interface science.

[B41] Cassell AM, Lee Asplund C, Tour JM (1999). Self-assembling supramolecular nanostructures from a C_60 _derivative: Nanorods and Vesicles. Angew Chem Int Ed.

